# Co-release of glutamate and GABA from single vesicles in GABAergic neurons exogenously expressing VGLUT3

**DOI:** 10.3389/fnsyn.2015.00016

**Published:** 2015-09-23

**Authors:** Johannes Zimmermann, Melissa A. Herman, Christian Rosenmund

**Affiliations:** Neurowissenschaftliches Forschungszentrum (NWFZ), NeuroCure Exzellenzcluster, CCO Charité UniversitätsmedizinBerlin, Germany

**Keywords:** co-release, vesicular transporter, synaptic transmission, synergy, VGLUT

## Abstract

The identity of the vesicle neurotransmitter transporter expressed by a neuron largely corresponds with the primary neurotransmitter that cell releases. However, the vesicular glutamate transporter subtype 3 (VGLUT3) is mainly expressed in non-glutamatergic neurons, including cholinergic, serotonergic, or GABAergic neurons. Though a functional role for glutamate release from these non-glutamatergic neurons has been demonstrated, the interplay between VGLUT3 and the neuron’s characteristic neurotransmitter transporter, particularly in the case of GABAergic neurons, at the synaptic and vesicular level is less clear. In this study, we explore how exogenous expression of VGLUT3 in striatal GABAergic neurons affects the packaging and release of glutamate and GABA in synaptic vesicles (SVs). We found that VGLUT3 expression in isolated, autaptic GABAergic neurons leads to action potential evoked release of glutamate. Under these conditions, glutamate and GABA could be packaged together in single vesicles release either spontaneously or asynchronously. However, the presence of glutamate in GABAergic vesicles did not affect uptake of GABA itself, suggesting a lack of synergy in vesicle filling for these transmitters. Finally, we found postsynaptic detection of glutamate released from GABAergic terminals difficult when *bona fide* glutamatergic synapses were present, suggesting that co-released glutamate cannot induce postsynaptic glutamate receptor clustering.

## Introduction

In synaptic transmission, postsynaptic responses are determined in large part by the identity of the neurotransmitter released at the presynaptic terminal, which is, in turn, determined by the type of vesicular neurotransmitter transporter expressed by the presynaptic neuron. The vesicular glutamate transporter 3 (VGLUT3) is expressed mainly in neurons that do not release glutamate as a primary transmitter, such as cholinergic, serotoninergic, or GABAergic neurons (Fremeau et al., 2002; Gras et al., 2002; Schäfer et al., 2002; Takamori et al., 2002). In cholinergic and serotonergic terminals, VGLUT3 stimulates the vesicular uptake of acetylcholine (Ach; Gras et al., 2008; Nelson et al., 2014) and serotonin (5-HT; Amilhon et al., 2010), respectively, to synaptic vesicles (SVs). This is likely because the co-packaging of glutamate, a negatively charged molecule, causes an increase in the pH gradient across the vesicular membrane, which then synergistically increases the driving force for uptake of the other neurotransmitters (Hnasko et al., 2010; El Mestikawy et al., 2011). However, the vesicular GABA transporter (VGAT) is thought to rely less on the pH gradient than the transmembrane electrical potential (Hell et al., 1990), which would not be enhanced by co-packaging of glutamate. Nevertheless, transport assays suggest evidence for synergistic effects for the uptake of GABA by glutamate (Zander et al., 2010), but these effects have not been investigated in SVs release from individual terminals.

In addition to potential synergistic roles for glutamate in loading of GABA into SVs, there is also the question of whether glutamate may play a functional role in postsynaptic signaling at a GABAergic synapse. It has been suggested that co-release of glutamate from GABAergic synapses contributes to tonotopic organization in the mouse auditory brain stem (Noh et al., 2010), and recently, that GABA/glutamate co-release affects the output of the lateral habenula, an area implicated in depression (Root et al., 2014; Shabel et al., 2014).

In synapses expressing both VGLUT and VGAT, are glutamate and GABA packaged into the same vesicles? There are hints that this is the case (Zander et al., 2010; Weston et al., 2011; Beltrán and Gutiérrez, 2012); however, existing studies have not provided quantitative analysis of the composition of individual quantal events. In this study we address whether GABA and glutamate can be stored in and released from the same vesicle by recording postsynaptic events in autaptic GABAergic neurons cultured from the striatum (Bekkers and Stevens, 1991) exogenously expressing VGLUT3. We found that action potentials in GABAergic neurons expressing VGLUT3 evoked mixed Postsynaptic currents (PSCs) mediated by both GABA and glutamate release. Analysis of the spontaneously released miniature PSC (mPSC) and asynchronous event kinetics suggest that the quantal events underlying the evoked mixed PSC included vesicles containing both glutamate and GABA; however, no change in the amount of GABA packaged into vesicles with or without VGLUT3 arguing against synergy for loading between these two neurotransmitters. Interestingly, glutamate co-released with GABA from GABAergic terminals was difficult to detect in cultures that contained *bona fide* glutamatergic synapses, likely because AMPA receptors in this case are sequestered away from the GABAergic postsynaptic sites. This indicates that, at least in GABAergic synapses exogenously expressing VGLUT3, glutamate release alone is not sufficient to cause clustering of postsynaptic AMPA receptors.

## Materials and Methods

### Neuronal Culture

All experiments involving animals were performed according to the regulations of the Animal Welfare Committee of the Charité Universitätsmedizin and the Berlin state government (permit # T0220/09).

Autaptic neuronal cultures were carried out as described previously (Xue et al., 2007). Briefly, WT C57/BL6N mice were sacrificed at P0–2. Brains were dissected out and placed in 4°C cooled HBSS. Hippocampus or striatum, respectively, were removed and incubated in a papain-containing solution to dissociate the cells. After 45 min the reaction was stopped by gently exchanging the solution to an inactivating solution containing 2.5 mg albumin/ml and 2.5 mg/ml trypsin-inhibitor (Sigma-Aldrich, Germany) in 5% fetal calf serum (FCS). After 5 min the medium was replaced by neuronal growth medium (neuronal basal A (NBA), supplemented with B27, Glutamax (Gibco Life Technologies, Germany), and penicillin/streptavidin (Roche, Germany)) and the cells were counted in a Neubauer counting chamber. For neuronal autaptic cultures 2,500 hippocampal or 3,000 striatal neurons were seeded onto astrocyte-layered microislands grown on 30 mm diameter coverslips. For mass cultures, 50,000 neurons were seeded per well on astrocyte-layered 12-well (22 mm diameter) plates. Neurons were grown for 9–16 days *in vitro* (DIVs) at 37°C in 5% CO_2_.

### Lentivirus Constructs and Production

Sequence of murine VGLUT3 was cloned into a lentiviral shuttle vector under the control of a human synapsin-1 promoter. To enable identification of infected cells the expression cassette of the protein was fused to a nuclear localization sequence-tagged green fluorescent protein (NLS-GFP) via a self-cleaving P2A peptide (Kim et al., 2011).

Lentiviral particles were prepared as described in Lois et al. (2002). HEK293T cells were cotransfected with 10 μg shuttle vector and the helper plasmids pCMVdR8.9 and pVSV.G (5 μg each) with X-tremeGENE 9 DNA transfection reagent (Roche Diagnostic, Germany). After 72 h the virus-containing cell culture supernatant was collected and purified by filtration. Aliquots were flash-frozen in liquid nitrogen and stored at −80°C. Virus efficiency was determined by titration with mice WT hippocampal mass-cultured neurons. For infection, about 1 × 10^6^ infectious particles were pipetted onto 1 DIV hippocampal neurons per 35 mm diameter well.

### Electrophysiology

Whole-cell patch clamp recordings were performed between DIV 9 and 16. The experiments were carried out at room temperature in an extracellular solution (ECS) containing the following (in mM): 140 NaCl, 2.4 KCl, 10 HEPES (Merck, Darmstadt, Germany), 10 glucose (Carl Roth, Karlsruhe, Germany), 2 CaCl_2_, (Sigma-Aldrich, St. Louis, USA), 4 MgCl_2_ (Carl Roth, Karlsruhe, Germany); 300 mOsm; pH 7.4. To block glutamatergic or GABAergic responses 10 μM 2, 3-Dioxo-6-nitro-1, 2, 3, 4-tetrahydrobenzo[f]quinoxaline-7-sulfonamide (NBQX; Tocris Bioscience, Bristol, UK) and 30 μM bicuculline (Bic; Tocris Bioscience, Bristol, UK), respectively, were added to the ECS. Internal solution contained the following (in mM): 136 KCl, 17.8 HEPES, 1 EGTA (Carl Roth, Karlsruhe, Germany), 4.6 MgCl_2_, 4 Na_2_ATP, 0.3 Na_2_GTP (Sigma-Aldrich, St. Louis, USA), 12 creatine phosphate (Calbiochem, Darmstadt, Germany), and 50 U/ml phosphocreatine kinase (Sigma-Aldrich, St. Louis, USA); 300 mOsm; pH 7.4.

Borosilicate glass pipettes (Science Products, Hofheim, Germany) had a resistance of 2–3.5 MΩ. All recordings were performed with a Multiclamp 700B amplifier and a Digidata 1440A digitizer under control of Clampex 10.0 (Molecular Devices, Sunnyvale, USA). Data was acquired at 10 kHz and filtered at 3 kHz. In most of the experiments, membrane capacitance and 70% of the series resistance were compensated while changes in series resistance were monitored frequently throughout the experiments. Only cells with a series resistance <10 MΩ were used for analysis. PSCs were elicited by a 2 ms somatic depolarization from −70 mV to 0 mV, which resulted in an unclamped action potential. Response amplitudes were measured from baseline. Quantal PSC (qPSC) were recorded as either spontaneously released events (miniature) or evoked in an ECS in which CaCl_2_ was replaced by SrCl_2_ (asynchronous). In both cases, GABAergic and glutamatergic components were pharmacologically isolated with NBQX or Bic, respectively. In order to optimize detection condition of the mPSCs, and because voltage escape poses less of a problem for single vesicle events, series resistance was uncompensated for analysis of mPSCs decay analysis (Figure 3). For analysis of asynchronous PSCs evoked in SrCl_2_ (Figure 4) series resistance was compensated to assure voltage control during the evoked component of the response.

### Analysis and Statistics

For analysis of qPSCs, events were detected from 1 kHz filtered traces by running a template-based detection algorithm in AxoGraph X 1.5.4 (AxoGraph, Berkeley, USA). To analyze the decay kinetics of quantal events for the co-release experiments we used two different methods, both resulting in a similar outcome: (1) After running the recognition template in AxoGraph the decays of detected mPSCs were fitted in AxoGraph with a single or double exponential using a simplex optimization procedure from the peak until the decay reached baseline. The fits were judged by eye and single exponential fits were discarded when clearly not representing the signal; and (2) Individual traces (from peak to baseline) were fitted in GraphPad Prism using a Levenberg-Marquardt algorithm and the software ran an *F* test to determine if a single or a double exponential was preferential. The decay time constants for each of the double exponential fits were converted to a weighted decay to compare to the single exponential decay time constant.

Gaussian fits of the decay distributions were performed in GraphPad Prism. The software ran an *F* test to determine if the data was fitted better by a single Gaussian or the sum of multiple Gaussians.

Comparison of means was performed in GraphPad Prism. After testing for normality with D’Agostino-Pearson normality test, the means of two groups were compared using the Student’s *t*-test or Mann-Whitney test (for non-parametric data). Three or more groups were compared using one-way ANOVA, followed by a Dunnett test.

## Results

### VGLUT3 Expression Induces Mixed GABA/Glutamate Evoked PSC in GABAergic Autapses

**Figure 1:**
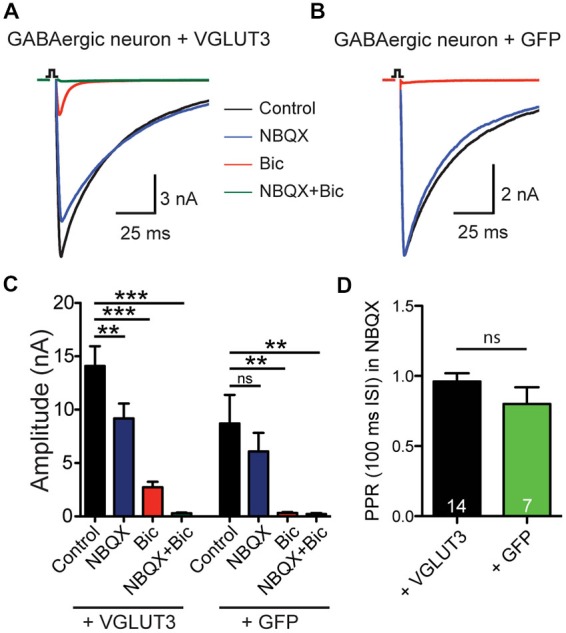
**VGLUT3 expression promotes glutamate release in GABAergic neurons. (A)** Exemplary traces of current responses to an unclamped AP of a striatal GABAergic neuron exogenously expressing VGLUT3 in control extracellular solution (ECS; black), the AMPA receptor antagonist, NBQX (blue), the GABA_A_ receptor antagonist, bicuculline (Bic; red), or both antagonists combined (green). **(B)** Example traces in the same conditions as **(A)** from a striatal GABAergic neuron expressing only GFP. Stimulations are indicated by an open square; stimulation artifacts and action potentials have been blanked for illustrative purposes. **(C)** Plot of mean amplitudes of evoked response in VGLUT3-expressing cells (left; *n* = 13) and control cells expressing GFP (right; *n* = 6) in presence of NBQX, Bic or no drug (control). Significance was assessed by comparing responses in control conditions to each pharmacological treatment using an ANOVA repeated measures with Dunnett’s multiple comparison tests. Error bars = SEM. **p ≤ 0.01. ***p ≤ 0.001. **(D)** Plot of mean paired-pulse ratio (PPR) in the presence of NBQX for expressing VGLUT3 or GFP only. Significance was assessed by Mann-Whitney test. Error bars = SEM.

To test whether expression of a glutamate transporter was sufficient to elicit GABA/glutamate co-release from a GABAergic neuron, we expressed VGLUT3 in autaptic cultures (Bekkers and Stevens, 1991) from striatum using a lentiviral expression system. Whole-cell patch clamp recordings showed reliable PSCs upon depolarization stimulus (Figure 1). Addition of the GABA receptor antagonist Bic to the ECS revealed a fast excitatory component (Figures 1A,C) that could be blocked by the AMPA receptor antagonist NBQX, which suggests that glutamate could be released from these GABAergic terminals. As expected, in autaptic striatal GABAergic neurons expressing only GFP, but no VGLUT3, PSC responses were blocked by application of Bic, leaving no NBQX-sensitive component (Figures 1B,C). On average, the NBQX-sensitive component was significantly higher when cells exogenously expressed VGLUT3 (Bic minus Bic + NBQX: VGLUT3 2.43 ± 0.54 *n*A, *n* = 13; GFP 0.09 ± 0.14 nA, *n* = 6; **p = 0.0018, Mann-Whitney test).

We next examined whether paired-pulse behavior in the GABAergic synapses was altered by the expression of VGLUT3. We found that on average the paired-pulse ratio (PPR), as assessed by the amplitude of a second stimulation evoked PSC divided by that of the first with an interpulse interval of 100 ms, of the purely GABAergic response was not different between cells that expressed VGLUT3 and those that did not (Figure 1D; *p* = 0.314, Mann-Whitney test). This suggests that co-release of glutamate from GABAergic neurons does not affect presynaptic release probability.

### Glutamate Release from GABAergic Autaptic Neurons does not Change AMPAR Expression

**Figure 2:**
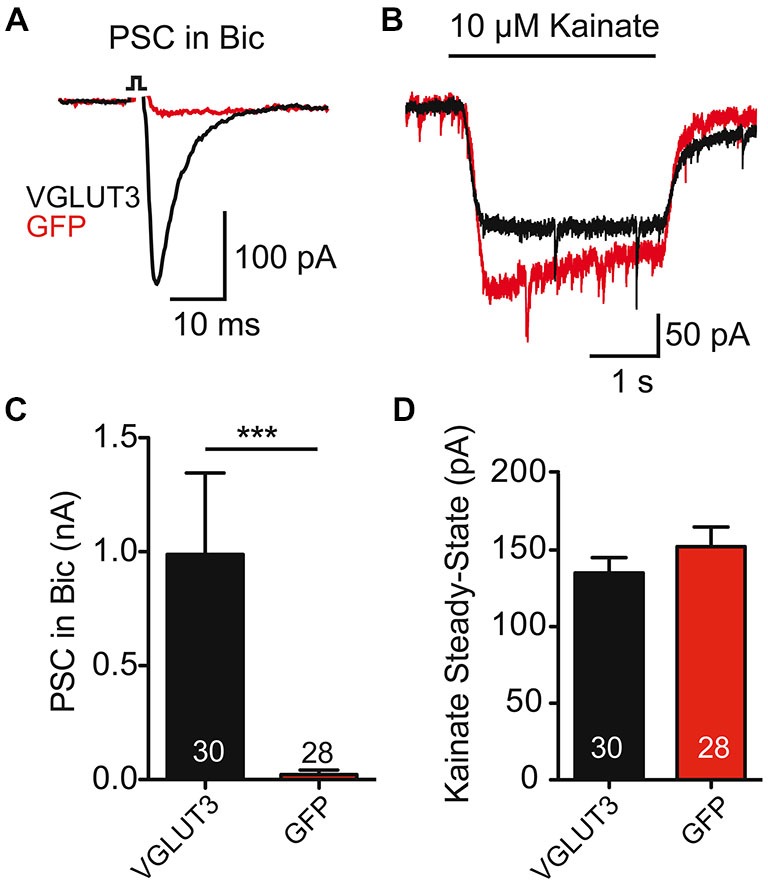
**AMPA receptor-mediated currents are unaltered in co-releasing neurons. (A)** Exemplary traces of Postsynaptic currents (PSCs) from striatal GABAergic neurons expressing VGLUT3 (black) or GFP (red) in the presence of Bic. **(B)** Responses to kainate (10 μM) application in the neurons from **(A)**. **(C)** Plot of average PSC (PSC) amplitude in presence of Bic. ***p < 0.0001. **(D)** Plot of average kainate steady-state current. Number of cells indicated in graphs. Error bars = SEM.

As striatal GABAergic neurons expressing VGLUT3 showed an evoked glutamate-mediated response, we next wondered whether glutamate release changed the expression of AMPA-type glutamate receptors on these cells. To test this, we compared the current evoked by application of kainate (10 μM) to striatal cells expressing VGLUT3 + GFP or GFP alone (Figure 2B). We found that kainate evoked a response in all cells, whether they expressed VGLUT3 or not (Figures 2B,D; *p* = 0.290, Student’s *t*-test); however, only cells expressing VGLUT3 had a glutamatergic synaptic response in the presence of Bic (30 μM; Figures 2A,C; *p* < 0.0001, Mann-Whitney test). Together, these data suggest that AMPA receptor expression on GABAergic autaptic neurons from striatum is not altered by co-release of GABA and glutamate.

### Mixed Glutamate/GABA Postsynaptic Responses Caused by Spontaneous Release in VGLUT3-Expressing Striatal Neurons

**Figure 3:**
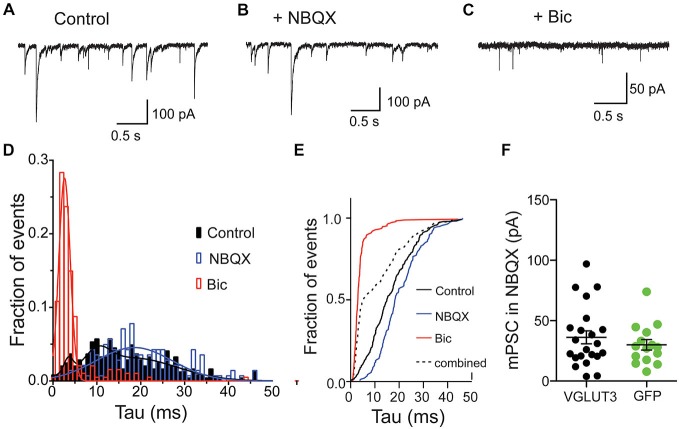
**Glutamate and GABA are released from the same vesicles but do not synergize.** Exemplary traces of spontaneous release in striatal autaptic neurons expressing VGLUT3 under three recording conditions: in control ECS **(A)**, ECS with NBQX **(B)** and ECS with Bic **(C)**. **(D)** Histogram of decay times (tau) of spontaneous events under the conditions described in **(A–C)** from 8 neurons. The plots were fitted with single Gaussian (NBQX and Bic; red and blue lines) or with the sum of three Gaussians (control; black line), respectively. **(E)** Cumulative frequency plot of decay times from histogram in **(D).** Values from “NBQX group” and “Bic group” were combined to create the “combined group”. **(F)** Comparison of mPSC amplitudes in presence of NBQX. Circles represent individual cells. Horizontal lines show mean ± SEM.

Spontaneous release of single vesicles is an ideal system to study if glutamate and GABA are stored in distinct vesicle pools or if they are stored in the same vesicles. We recorded miniature postsynaptic events (mPSCs) from VGLUT3-expressing, autaptic, striatal neurons in control ECS without antagonists and in the presence of NBQX or Bic to isolate a GABAergic or glutamatergic component of the response, respectively (Figures 3A–C). Similar to our results with evoked release we observed fast-decaying events in the presence of Bic (Figure 3C) and slower-decaying events in the presence of NBQX (Figure 3B). To determine whether the events in the control solution simply represent the sum of the events in NBQX and Bic or if there is also a population of mixed events, we pooled the events from each condition and analyzed the decay times. We found that distributions for all of these populations were significantly different (*p* < 0.0001 for all combinations; Kolmogorov-Smirnov test). The frequency histograms for events recorded in Bic or NBQX were best fitted with a single Gaussian (Figure 3D; Bic: mean tau ± SD, 2.6 ± 1.3 ms, *R*^2^ = 0.96; NBQX: mean tau ± SD, 18.5 ± 8.7 ms, *R*^2^ = 0.68). The frequency histogram for events recorded in control solution was best fitted by the sum of three Gaussians (mean tau1 ± SD1, 3.5 ± 1.0 ms, mean tau2 ± SD2, 10.2 ± 2.8 ms, mean tau3 ± SD3, 17.8 ± 10.3 ms *R*^2^ = 0.86).

To confirm that these events recorded in the control conditions actually contained a population of mixed glutamate-GABA events, we compared the cumulative frequency distribution of the control events to an artificial population consisting of a combination of events recorded in Bic or NBQX (Figure 3E). We found that the distribution of these two populations was significantly different (*p* < 0.0001, Kolmogorov-Smirnov test; Figure 3E), suggesting that a significant portion of spontaneous events recorded from VGLUT3-expressing GABAergic neurons reflect co-release of glutamate and GABA from the same synaptic vesicle.

We next tested whether co-packaging of glutamate and GABA leads to an increase in GABA content in single vesicles. This synergistic effect was found in other dual-transmitter systems (Amilhon et al., 2010; Hnasko et al., 2010). We compared the GABA content of minis by measuring spontaneous event amplitudes in the presence of NBQX. Striatal interneurons expressing VGLUT3 did not show altered mini amplitudes compared to control interneurons expressing GFP (Figure 3F). Thus, the presence of glutamate in those vesicles does not seem to have a synergistic effect on GABA uptake.

### Glutamate and GABA Containing Vesicles are Released by AP Stimulation

**Figure 4:**
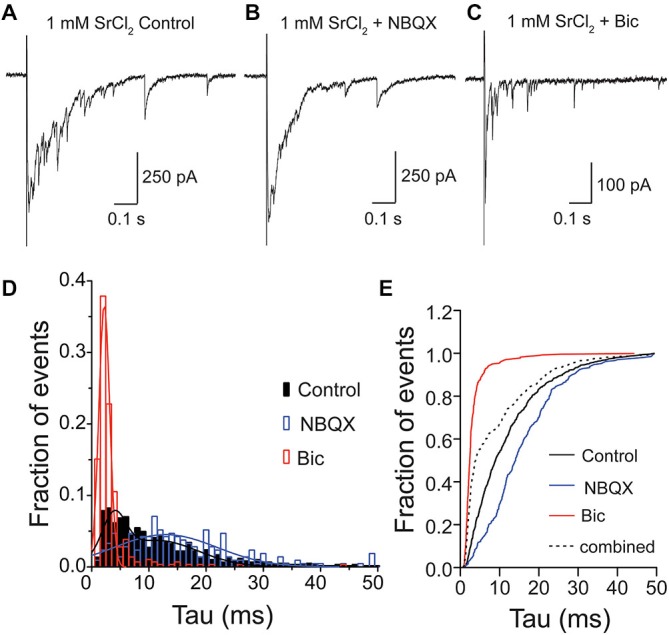
**Glutamate and GABA are released from the same vesicles during evoked asynchronous release.** Exemplary traces of Sr^2+^-evoked asynchronous release in striatal autaptic neurons expressing VGLUT3 under three recording conditions: in control ECS **(A)** ECS with NBQX **(B)** and ECS with Bic **(C)**. **(D)** Histogram of decay times (tau) of quantal events under the conditions described in **(A–C)**. Events were pooled from 11 cells. The plots were fitted with single Gaussian (NBQX and Bic; red and blue lines) or with the sum of two Gaussians (control; black line), respectively. **(E)** Cumulative frequency plot of decay times. Values from “NBQX group” and “Bic group” were combined to create the “combined group”.

For many years, it has been debated whether spontaneous or stimulus-evoked neurotransmitter release modes utilize vesicles from the same pool (Sara et al., 2005; Groemer and Klingauf, 2007). Therefore, we wanted to test whether, in addition to spontaneously released vesicles containing both GABA and glutamate in the VGLUT3-expressing GABAergic neurons (Figure 3), vesicles released by stimulation could also contain both transmitters. To address this, we recorded from VGLUT3-expressing cultured, autaptic striatal neurons. To study the content of single, evoked vesicles, we replaced the extracellular Ca^2+^ with Sr^2+^ (1 mM), and pharmacologically dissected the responses under these conditions (Figure 4). As expected (Goda and Stevens, 1994), the addition of [Sr^2+^]_e_ produced asynchronous events, including putative qPSC events (Figures 4A–C). We examined the neurotransmitter content of these quantal events (single peak events that occurred within one spost-stimulus) in control ECS, with the addition of NBQX or Bic (Figures 4A–C), and pooled the asynchronous events from eleven cells, which were both GFP positive and displayed detectable events in the presence of Bic. When all events were pooled from each condition, we found that distributions for each of these populations were significantly different (*p* < 0.0001 for all combinations; Kolmogorov-Smirnov test). The frequency histograms for events recorded in Bic or NBQX were best fitted with a single Gaussian (Figure 4D; Bic: mean tau ± SD, 2.2 ± 0.9 ms, *R*^2^ = 0.97; NBQX: mean tau ± SD, 12.9 ± 9 ms, *R*^2^ = 0.77). The frequency histogram for events recorded in control ECS was best fitted by the sum of two Gaussians, where the mean amplitude of each Gaussian peak was shifted to the right or left of the Bic or NBQX isolated events, respectively (mean tau1 ± SD1, 3.9 ± 2 ms, mean tau2 ± SD2, 11.4 ± 7.2 ms, *R*^2^ = 0.87). To confirm that the events recorded in the control extracellular conditions contained a population of mixed glutamate-GABA events, we compared the cumulative frequency distribution of the control events to an artificial population consisting of a combination of events recorded in Bic or NBQX (Figure 4E). We found that the distribution of these two populations was significantly different (*p* < 0.0001, Kolmogorov-Smirnov test; Figure 4E), suggesting that a portion of asynchronous events recorded from VGLUT3-expressing GABAergic neurons reflect co-release of glutamate and GABA from the same synaptic vesicle. It should be noted that the decay times for GABA only and glutamate/GABA mixed asynchronous qPSCs were faster than those reported for mPSCs. This is likely because the strontium recordings were performed with series resistance compensation (see Section “Materials and Methods”).

### Glutamate/GABA Co-Release in a Developing Circuit

**Figure 5:**
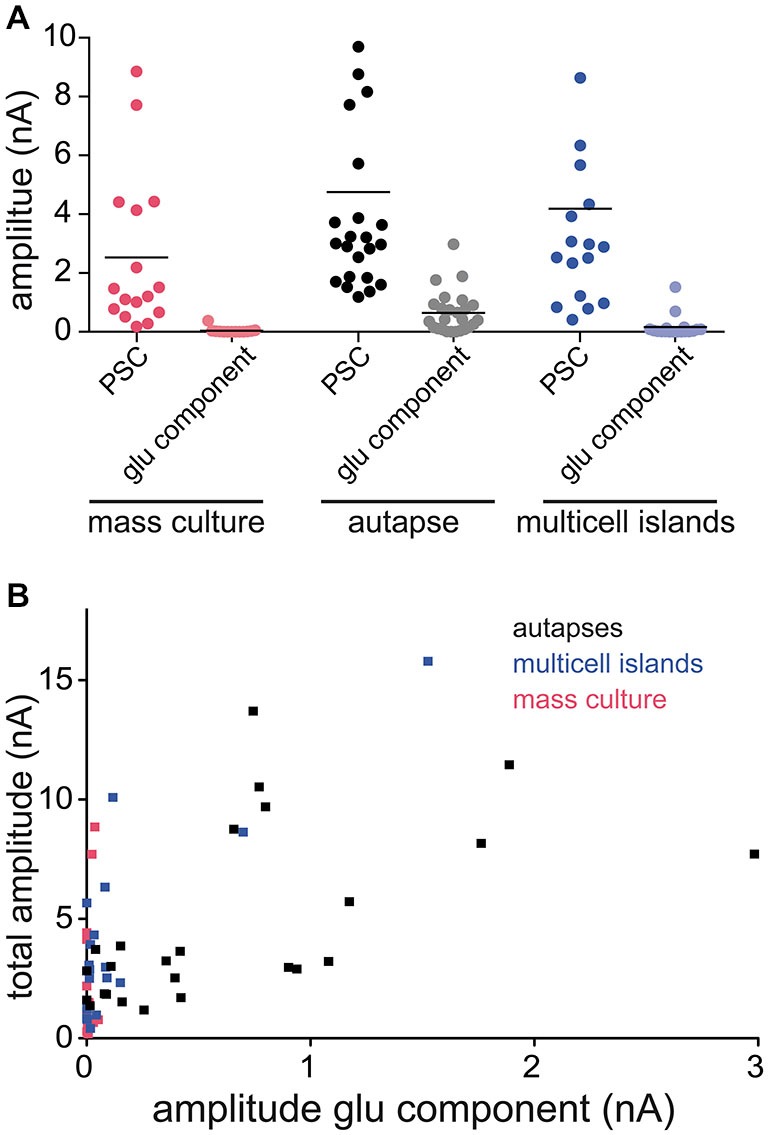
**Amount of detectable glutamate co-release varies between different culture systems. (A)** Plot of PSC amplitudes in GABAergic striatal neurons expressing VGLUT3. Recordings in three different culture systems: mass culture (red), autaptic culture (black/gray) and 2 or more cells on an astrocyte feeder island (blue). Depicted are the PSC sizes in control ECS (darker colors) and the glutamatergic component in the presence of Bic (lighter colors). Each data point represents a cell. Horizontal lines show the average response size. **(B)** Plot of PSC amplitude in ECS (“total amplitude”) against amplitude in Bic (“amplitude glu component”) of the same cell compared over the three different culture systems.

Our findings show that glutamate can be both released and detected at GABAergic synapses from autapses expressing VGLUT3. However, in neural circuits in the brain, GABAergic synapses co-releasing glutamate must exist on the same postsynaptic target cells as *bona fide* glutamatergic synapses. Therefore, we examined whether we could still detect glutamate released from GABAergic striatal neurons expressing VGLUT3 when they were cultured in the presence of glutamatergic neurons from hippocampus. We performed paired whole cells recordings from mixed cultures of striatal and hippocampal neurons infected with VGLUT3, and pharmacologically isolated the PSCs as previously described (Figures 1, 3–4). On average, the responses from VGLUT3-expressing GABAergic presynaptic neurons were almost completely blocked by application of Bic, indicating that glutamatergic co-release could not be detected. To test whether the lack of detection of glutamate co-release from GABAergic neurons was due to a low expression level of VGLUT3 in mixed striatal/hippocampal cultures, we performed sister-culture experiments with pure striatal neurons or mixed striatal/hippocampal neurons plated on astrocyte microislands, and again recorded from autaptic striatal neurons expressing VGLUT3 or from paired neurons in multi-cell microisland circuits. We found, again, that the majority of autaptic GABAergic neurons expressing VGLUT3 had an NBQX-sensitive component of their evoked release (Figure 5A). On the other hand, the PSCs detected in paired recordings from multi-cell microislands where striatal GABAergic neurons expressing VGLUT3 were co-cultured with hippocampal neurons were almost entirely blocked by Bic application. Plotting the total amplitude of each cell against its amplitude in the presence of Bic (“glutamatergic component”) demonstrates the influence of the culturing system on the size of the glutamatergic component compared to the influence of the total response size (Figure 5B). While this could indicate that VGLUT3 is actively excluded from GABAergic vesicles in mixed cultures, a more likely explanation could be that even though glutamate is co-released from GABAergic terminals expressing VGLUT3, the detection could be limited by the availability of postsynaptic AMPA receptors, which may be sequestered by *bona fide* glutamatergic synapses in a network environment.

## Discussion

Co-release of two or more classical neurotransmitters from the same neuron is now considered to be a common principle throughout the nervous system (Whittaker et al., 1972; Jonas et al., 1998; Higley et al., 2011; Beltrán and Gutiérrez, 2012). We studied the co-release of glutamate and GABA in striatal interneurons, which exogenously expressed VGLUT3, and present the following findings: (1) Striatal GABAergic SVs do not have active mechanisms to exclude vesicular glutamate transporters and the presence of VGLUT on these vesicles is enough to induce glutamate co-release; (2) Glutamate and GABA are at least partially stored in the same vesicles and these vesicles are involved in both spontaneous and evoked (asynchronous) release; (3) Co-storage of glutamate and GABA does not result in neurotransmitter synergy as GABA content is unaltered in VGLUT3-expressing vesicles; and (4) In a network of glutamatergic and GABAergic neurons co-release of glutamate is not sufficient to induce clustering of AMPA receptors at the GABAergic postsynaptic structure.

Pharmacological dissection of evoked PSCs from GABAergic neurons expressing the VGLUT3 transporter has shown that these responses contain both a glutamatergic and GABAergic component (Figure 1; Weston et al., 2011). However, these measurements cannot discern between glutamate and GABA released from the same or separate synapses or vesicles. Therefore, to further investigate this question, we took advantage of the distinct kinetics of postsynaptic responses mediated by GABA_A_ and AMPA receptors (Figure 3). We found that a portion of spontaneously released mPSCs in striatal autapses expressing VGLUT3 showed a postsynaptic response, the decay of which was a convolution of the kinetics of GABA_A_ and AMPA receptor responses, suggesting that glutamate and GABA are released from the same vesicle. Importantly, we could also show that co-release of glutamate and GABA can occur in response to presynaptic stimulation, as asynchronous PSC evoked in the presence of SrCl_2_ also contained a population with mixed GABA_A_ and AMPA receptor kinetics (Figure 4). It should be noted that the percentage of mixed events detected from the total population of spontaneous or asynchronous events does not represent the fraction of vesicles that contain glutamate and GABA *per se* as the composition of postsynaptic receptors is unlikely to be uniform.

Does the co-storage of glutamate in GABAergic vesicles have synergistic effects on filling? Our findings and a recent study by Case et al. (2014), indicate that GABA content is not influenced by co-packaging of glutamate (but see Zander et al., 2010). A series of studies show that the presence of VGLUT3 in cholinergic and serotonergic terminals stimulated the uptake of ACh and serotonin (5-HT) to SVs (Gras et al., 2008; Amilhon et al., 2010). These so called “synergistic effects” have been attributed to an increase in the pH gradient (ΔpH) across the vesicle membrane due to the presence of glutamate, which then drives neurotransmitter uptake (Hnasko et al., 2010; El Mestikawy et al., 2011). However, neurotransmitter transport by the vesicular acetylcholine transporter (VAChT) and the vesicular monoamine transporter (VMAT2) into SVs depends largely on ΔpH (Johnson et al., 1981; Nguyen et al., 1998). Because accumulation of GABA by VGAT depends both on the transmembrane potential (Δψ) and on ΔpH (Hell et al., 1990), the acidification of SVs by VGLUTs seems to be less important than in the case of the positively-charged ACh, dopamine and 5-HT.

Even though multiple cell types in the brain have been identified where vesicular GABA and glutamate transporters are co-expressed, it has been difficult to prove co-release of GABA and glutamate at those synapses. Co-release of the two fast-acting NTs has been shown in the lateral superior olive (LSO; Gillespie et al., 2005) and in hippocampal mossy fibers (Gutiérrez, 2003; Beltrán and Gutiérrez, 2012). GABA/glycinergic synapses from the medial nucleus of the trapezoid body (MNTB) express VGLUT3 and co-release glutamate, which is important for the refinement of an inhibitory map in the auditory system (Noh et al., 2010). It has also been shown that glutamatergic granule cells in the dentate gyrus transiently release GABA during development from single mossy fiber giant boutons in hippocampal brain slice (Beltrán and Gutiérrez, 2012; Münster-Wandowski et al., 2013) and in autaptic culture (Valente et al., 2015).

One important question from a cell biological standpoint is whether co-release of glutamate at a GABAergic synapse influences the postsynaptic receptor identity. We found that in isolated, autaptic GABAergic neurons cultured from striatum, expression of VGLUT3 induced co-release of glutamate detected postsynaptically at the same synapses as GABA (Figures 3–4). This suggests that GABAergic synapses in the autaptic model contain AMPA receptors. Interestingly, it has been shown that GABAergic neurons grown in isolation have a diffuse distribution of AMPA receptors throughout their dendrites; however, in the presence of glutamatergic input, AMPA receptor distribution becomes punctate (Rao et al., 2000). We found that in the presence of glutamatergic input in mass culture or multicellular microislands, co-release of glutamate from GABAergic terminals was no longer detectable through AMPA receptor-mediated currents (Figure 5). Because our experiments comparing co-release in isolated, GABAergic striatal neurons and GABAergic striatal neurons in mixed mass culture used sister cultures expressing VGLUT3 from the same constructs, we speculate that the lack of AMPA receptor-mediated currents in only the mass culture model is not due to lack of VGLUT3 expression in these cells. Instead, we favor the interpretation that with glutamatergic input, AMPA receptors are recruited to glutamatergic synapses, and that co-release of glutamate from a GABAergic terminal is not sufficient to induce AMPA receptor clustering at its postsynaptic element. However, it cannot be ruled out that at GABAergic synapses endogenously co-expressing VGLUT3, that the postsynaptic element may contain specific molecules promoting the capture of AMPA receptors.

We have shown, as a proof of principle, that on a quantal level, expression of both VGLUT and VGAT in a presynaptic terminal leads to glutamate/GABA co-release. How might this co-release influence signaling at VGLUT3-expressing GABAergic terminals? It is feasible that co-released glutamate could act through a spillover mechanism to activate metabotropic glutamate receptors (mGluRs), which are present on GABAergic terminals (Somogyi et al., 2003). Another distinct possibility is that glutamate may activate high-affinity NMDA receptors, which have been suggested to exist at VGLUT3-expressing GABAergic terminals in adult rat hippocampus (Stensrud et al., 2015). In addition, activation of NMDA receptors by co-released glutamate has been proposed as the mechanism by which expression of VGLUT3 in GABAergic synapses refines the tontopic map in auditory brainstem (Gillespie et al., 2005; Noh et al., 2010). Further details regarding how glutamate co-release at GABAergic terminals in various regions with endogenous VGLUT3 expression affects signaling at the synaptic level and function at the circuit level are interesting questions to be addressed by further studies.

## Funding

This work was supported by the Boehringer Ingelheim Fonds (PhD fellowship to JZ) and the German Research Council (DFG Training Grant GRK1123 to JZ), the European Research Foundation (Grant SynVglut to CR), and the Excellence Cluster NeuroCure Exc257 (to CR).

## Conflict of Interest Statement

The authors declare that the research was conducted in the absence of any commercial or financial relationships that could be construed as a potential conflict of interest.
